# A dataset of synthetic hexagonal close packed 3D polycrystalline microstructures, grain-wise microstructural descriptors and grain averaged stress fields under uniaxial tensile deformation for two sets of constitutive parameters

**DOI:** 10.1016/j.dib.2018.10.172

**Published:** 2018-11-03

**Authors:** Ankita Mangal, Elizabeth A. Holm

**Affiliations:** Carnegie Mellon University, United States

## Abstract

This data article presents a data set comprised of 54 synthetic 3D equiaxed polycrystalline microstructures, the microstructural descriptors for each grain and the stress fields resulting from two sets of crystal plasticity simulations mimicking uniaxial tensile deformation to a total strain of 2%. This is related to the research article entitled “Applied Machine Learning to predict stress hotspots II: Hexagonal Close Packed Materials” (Mangal and Holm, 2018). The microstructures were created using an open source Dream.3D software tool and the crystal plasticity simulations were carried out using elasto-viscoplastic fast Fourier transform (EVPFFT) method. Eight different kinds of HCP textures are represented with stochastically different microstructures with varying texture intensity for each texture kind. For each texture kind, between six and nine stochastically different microstructures with varying texture intensity (measured by multiples of random density (MRD)) are created. This dataset is freely available in two Mendeley Data archives “Synthetic HCP 3D polycrystalline microstructures with grain-wise microstructural descriptors and stress fields under uniaxial tensile deformation: Part One” and “Synthetic HCP 3D polycrystalline microstructures with grain-wise microstructural descriptors and stress fields under uniaxial tensile deformation: Part Two” located at http://dx.doi.org/10.17632/kt8hfg4t2p.1 and http://dx.doi.org/10.17632/nsfn6tw295.1 respectively for any academic, educational, or research purposes.

**Specifications table**

**Table:** 

Subject area	*Materials Science*
More specific subject area	*Mechanical Metallurgy, Crystal Plasticity*
Type of data	*3D synthetic microstructures, Voxel-wise and grain-wise stress field, slip activities, Taylor factors and microstructural descriptors, scripts*
How data was acquired	*3D microstructures were created using open source Dream.3D software*[2]*, Constitutive Modeling was done using EVPFFT*[3]
Data format	*Processed digital 3D microstructures in.HDF5 format, formatted scripts in.HTML format*
Experimental factors	*Microstructures are created at a resolution of 128 × 128 × 128 voxels, consisting around 5000 grains each, with a lognormal grain size distribution and a mean grain size of 2.7 μm.*
	*The constitutive parameters represent two materials: 1) A generic α-Titanium alloy*[4]*with basal <a>, prismatic <a> and pyramidal <c+a> slip systems with critically resolved shear stress (CRSS) ratio of 0.7:1:3 respectively and 2) a hypothetical HCP material with basal <a>, prismatic <a>, pyramidal <a> and pyramidal <c+a> slip systems with equal CRSS ratio between the deformation modes.*
Experimental features	*The Voce Hardening model was used to describe the strain hardening behavior during tensile deformation*
	*The boundary conditions for EVPFFT crystal plasticity simulations correspond to uniaxial tension along Z, with an applied strain rate component along the tensile axis ε ˙*_*33*_*= 1 s*^*−1*^*. The simulation was carried out in 200 steps of 0.01%, up to a strain of 2% for hypothetical HCP material with equal CRSS ratio and 100 steps of 0.01%, up to a strain of 1% for generic α-Titanium alloy with unequal CRSS ratio.*
Data source location	*Pittsburgh, PA, USA*
Data accessibility	*Data are publicly available via Mendeley Data at*http://dx.doi.org/10.17632/kt8hfg4t2p.1[5] for *the hypothetical HCP material parameters (Equal CRSS ratio case) and at*http://dx.doi.org/10.17632/nsfn6tw295.1[6] for the *generic α-Titanium alloy*[4]*parameters (UnEqual CRSS ratio case)*
Related research article	*Dataset used in “Applied Machine Learning to predict stress hotspots II: Hexagonal Close Packed Materials” (Mangal and Holm, 2018)**[1]*

**Value of the data**

• This simulated dataset provides a large number of microstructures to analyze relationships between the microstructure and stress and strain fields.
• The dataset is valuable for developing and benchmarking data driven techniques for applications which require data describing microstructure, stress and strain fields and corresponding slip activities in grains such as analyzing microstructure-property relationships in the context of material failure.
• The 3D microstructures can be used to train machine learning algorithms for identifying grain size distribution from images.
• The dataset can be used to understand and compare crystal plasticity relationships in HCP materials with contrasting constitutive parameters. The data may also be compared with the tensile behavior of other HCP materials.
• The dataset can be used to pre-train machine learning models for transfer learning on experimentally obtained high energy electron diffraction (HEDM) measurements.

## 1 Data

**Fig. 1:**
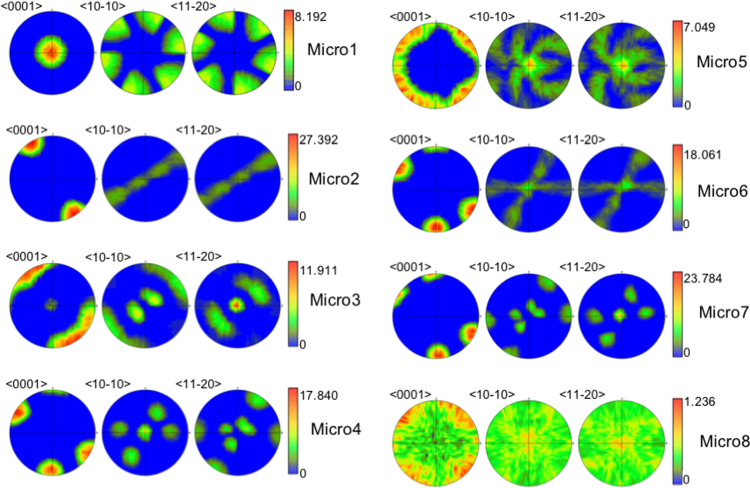
Representative pole figures for 8 different kinds of HCP textures chosen in this data.

This data set consists of 54 synthetic 3D equiaxed polycrystalline microstructures with different hexagonal close packed textures, associated microstructural descriptors and the corresponding results from applying a uniaxial tensile stress through EVPFFT simulations. The microstructures are divided into 8 major texture classes, whose representative textures are shown in Fig. 1. The EVPFFT simulations are carried out using constitutive parameters listed in Table 1, Table 2.

**Table 1:** Single crystal elastic stiffness constants (in GPa).

**C**_**11**_	**C**_**12**_	**C**_**13**_	**C**_**33**_	**C**_**44**_	**C**_**66**_
170	98	86	204	51	66

**Table 2:** Voce hardening parameters imitating Titanium and a hypothetical HCP material.

**Material**	**CRSS ratio**	**Slip Systems**	$\tau_{0}^{s}$**(MPa)**	$\tau_{1}^{s}$**(MPa)**	$\theta_{0}^{s}$	$\theta_{1}^{s}$
***α-Titanium***	0.7:1:3	Basal <a>	82.8	36.7	406.3	4.6
		Prismatic <a>	57.9	25.7		
		Pyramidal <c+a>	248.5	110.1		
***Ideal HCP***	1:1:1	Basal <a>	100	50	500	10
		Prismatic <a>				
		Pyramidal <c+a>				
		Pyramidal <c+a>				

## 2 Experimental design, materials, and methods

### 2.1 Synthetic microstructure generation pipeline

A dataset of synthetic microstructure images is built to be used as input to a parallelized elasto-viscoplastic Fast Fourier Transform (EVPFFT) crystal plasticity formulation for simulating a uniaxial tensile test. Dream.3D [2], [7] is used to create synthetic three-dimensional polycrystalline microstructures mimicking metallic alloys with equiaxed grains. The microstructure is generated from a set of defined statistics about grain size and texture distributions. These statistics can be defined using the Stats Generator filter inside Dream.3D. The microstructure is discretized on a 128 × 128 × 128 grid, which allows the use of image based crystal plasticity models.

The grain size distribution parameters used in the DREAM.3D Stats Generator are listed in Table 3. The Euler angles used to generate the eight representative textures are listed in Table 4. The texture intensity was varied to get between six and nine different instantiations within each representative texture. The eight crystallographic textures are represented as pole figures in Fig. 1. Overall, a total of 54 3D microstructures are included in the data set: between six and nine each of the eight textures.

**Table 3:** Dream.3D parameters for generating grain size distributions.

**Phase type:**	Primary	**Grain Shape**	Equiaxed
**Mean Grain Size (μ)**	2.3 μm	**Standard deviation (*σ*)**	0.4 μm
**Bin Step Size**	10	**Bins created**	4
**Max Grain Size cutoff**	*μ* + 3*σ*	**Min Grain Size cutoff**	*μ* − 4*σ*

**Table 4:** Bunge Euler angles (*ϕ*1, *θ*, *ϕ*2) used to generate the eight representative textures.

	*ϕ*1	*θ*	*ϕ*2		*ϕ*1	θ	ϕ2
Micro1	90	0	0	Micro5	0–90	90	90
Micro2	0–180	90	0–180	Micro6	0 or 60	90	90
Micro3	30	90	0–90	Micro7	0 or 60	90	0
Micro4	0 or 60	90	0–90	Micro8	n/a	n/a	n/a

### 2.2 Crystal plasticity simulations (micromechanical modeling)

We use an elasto-viscoplastic model based on fast Fourier transforms (EVPFFT) [3], [8], [9] to calculate the local stress and strain fields that develop in these synthetic microstructures when they are subjected to a uniaxial tensile deformation. The strain was chosen such that materials would transition from elastic into the plastic regime. Two cases are considered based on the strength of different slip systems i.e. having *Equal CRSS* and *Unequal CRSS* ratios. The CRSS ratio is defined with respect to the basal slip resolved shear strength ( $\tau_{\mathrm{basal}}$) as:

$$\mathrm{CRSS ratio}=\frac{\tau_{\mathrm{prismatic}<a>}}{\tau_{\mathrm{basal}<a>}}:1:\frac{\tau_{\mathrm{pyramidal}<c+a>}}{\tau_{\mathrm{basal}<a>}}:\frac{\tau_{\mathrm{pyramidal}<a>}}{\tau_{\mathrm{basal}<a>}}$$

In the elastic regime, the constitutive model parameters for both the HCP materials are similar to a general alpha-titanium alloy having an equiaxed microstructure [10]. The single crystal elastic constants are given in Table 1. The Equal CRSS case is hypothetical and is analyzed purely for model development and analysis. In this case, the deformation happens via 4 slip systems: basal<a>, prismatic<a>, pyramidal <a> and pyramidal<c + a> and the critically resolved shear stress (CRSS) is equal between deformation modes. The Unequal CRSS case representing an α-Titanium alloy has the CRSS ratio of 1: 0.7 : 3 between basal<a>: prismatic<a>: pyramidal<c + a> as shown by [11].

**Fig. 2:**
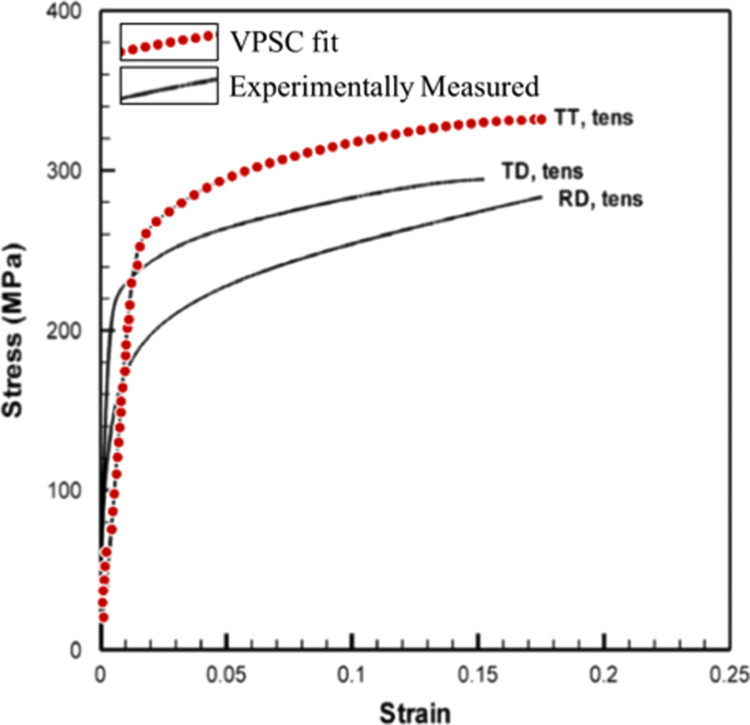
VPSC simulation fit to the experimentally observed stress-strain curve for alpha-Titanium. Reprinted from [1].

To obtain the actual CRSS values and the Voce hardening parameters, the Voce model was fit to an experimentally measured stress- strain curve for uniaxial tension in α-Titanium [12] using the VPSC formulation. The results of the fitting are shown in Fig. 2 and Table 2 lists the CRSS values and hardening parameters obtained for each CRSS ratio. Note that, for HCP materials; we have used 8 different kinds of textures summarized in Fig. 1. The stress exponent is 10 for all cases. The boundary conditions correspond to uniaxial tension along Z, with an applied strain rate component along the tensile axis ε ˙_33_ = 1 s^−1^. The EVPFFT simulation was carried out in 100 steps of 0.01%, up to a strain of 1% for Equal CRSS case and in 200 steps of 0.01%, up to a strain of 2% for Unequal CRSS case. The EVPFFT code package can be obtained by contacting the Richard P. Feynman Center for Innovation at Los Alamos National Laboratory [13].

### 2.3 Microstructural descriptors

**Fig. 3:**
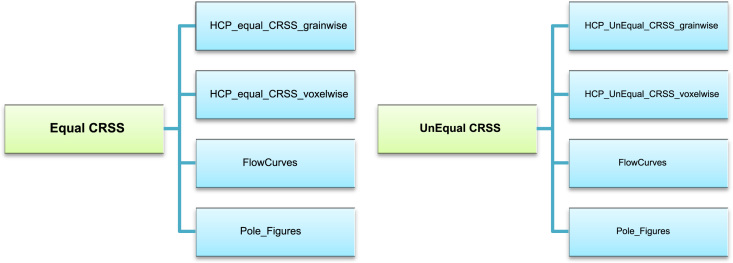
Dataset Folder Structure for the Mendeley archives “Synthetic HCP 3D polycrystalline microstructures with grain-wise microstructural descriptors and stress fields under uniaxial tensile deformation: Part One” and “Synthetic HCP 3D polycrystalline microstructures with grain-wise microstructural descriptors and stress fields under uniaxial tensile deformation: Part Two” respectively.

For enabling machine learning, we constructed a set of crystallography and geometry based microstructural descriptors. Dream.3D filters provide a convenient way to calculate a number of microstructural descriptors. Table 5 describes how the crystallographic descriptors were computed. Table 6 describes how the geometry based descriptors were computed. The processed grain-wise descriptors and grain-wise EVPFFT simulation results are contained in the HCP grainwise folders. Fig. 3 and Table 7 describe the data contained in these files.

**Table 5:** Computing crystallographic descriptors.

**Feature type**	**Measured by:**
Distance from Inverse pole figure corners:	This distance measures the orientation of the sample direction with respect to the [001, 101] and [111] crystal directions. This is calculated from grain Euler angles using a custom function included in the script.
Average Misorientation	A Dream.3D filter “Find Feature Neighbor Misorientations” is used to calculate the list of misorientations between a grain and its nearest neighbors. This list is averaged to get the metric.
Schmid Factor	The Schmid factors of each slip system: basal<a>, prismatic<a>, pyramidal <a> and pyramidal<c + a> are calculated manually using the function Calc_Schmid_factors included in the python script.
HCP c axis orientation	A Dream.3D filter “FindAvgCAxes” is used to calculate the location of the c-axis in the sample reference frame and store it as unit vector components. This vector is transformed into Polar coordinates to create another set of c-axis descriptors.

**Table 6:** Computing geometry descriptors.

**Feature type**	**Measured by:**
Grain Size measurements	Different metrics of grain size such as equivalent spherical diameter, number of voxels in a grain and number of neighbors are calculated using the following Dream.3D filters: “Find Feature Sizes” and “Find Feature Neighbors”
Grain shape parameters	The grain shapes are measured using their aspect ratios and surface area to volume ratio. These properties are calculated using to the Dream.3D filters “Find Feature Shapes” and “Find Surface Area to Volume”
Shape averaged distance from special points	The Dream.3D filter “Find Euclidean Distance Map” computes the distance of each voxel from its nearest grain boundary, triple junction and quadruple point. The voxels belonging to each grain are averaged to get a shape averaged distance from the special points (grain boundary, triple junction and quadruple point).

**Table 7:** Data element descriptions.

**Data element**	**Description**	
**Directory**		
**HCP_voxelwise**	Contains voxel-wise representation of microstructure, stress, and strain field. 8 kind of textures, between six and nine stochastic instantiations per texture.	
**Files**		
micro1_1_voxel.hdf5	**Dataset keys:**	**Remarks:**
micro1_2_voxel.hdf5	EulerAngles	Voxel-wise Euler Angles (Kocks convention)
.		
.	ngr	Voxel-wise Grain IDs
.		
	DistanceFrom	Voxel-wise distance from grain boundaries, triple junctions and quadruple points
micro1_6_voxel.hdf5		
.		
	Taylor	Taylor factor calculated by EVPFFT
.		
.	XYZ	Location of the voxel in 3D volume of size 128×128×128
micro8_9_voxel.hdf5		
	strains	Voxel-wise components of the strain tensor: [׳e11׳, ׳e22׳, ׳e33׳, ׳e23׳, ׳e13׳, ׳e12׳]
	stresses	Voxel-wise components of the stress tensor: [׳str11׳, ׳str22׳, ׳str33׳, ׳str23׳, ׳str13׳, ׳str12׳]
**Directory**		
**HCP_grainwise**	Contains the processed data having grain-wise representation of microstructure, microstructural features, equivalent von mises stress, and strain field	
**Files**		
micro1_all_grainwise.csv	**Dataset keys:**	**Remarks:**
.	[‘fileID’]	Identifier within each texture kind
.		
	[‘ngr’]	Grain ID
micro2_all_grainwise.csv		
.	[׳x׳, ׳y׳, ׳z׳]	Grain centroid location
.		
.	[׳001_IPF_0׳, ׳001_IPF_1׳, ׳001_IPF_2׳]	Distance of the [001] sample direction from the 3 corners of the inverse pole figure as described in Mangal and Holm [14].
.		
micro4_all_grainwise.csv		
.	[׳100_IPF_0׳, ׳100_IPF_1׳, ׳100_IPF_2׳]	Distance of the [100] sample direction from the 3 corners of the inverse pole figure as described in Mangal and Holm [14].
.		
micro6_all_grainwise.csv		
	[׳010_IPF_0׳, ׳010_IPF_1׳, ׳010_IPF_2׳]	Distance of the [111] sample direction from the 3 corners of the inverse pole figure as described in Mangal and Holm [14].
.		
.		
	[׳EqVonMisesStress׳, ‘EqVonMisesStrain’]	Equivalent Von Mises Stress and Strain in the grain
micro8_all_grainwise.csv		
	[‘hotspot’]	True if the grain is a stress hotspot
	[׳euler_1׳, ׳euler_2׳, ׳euler_3׳]	Grain-wise Bunge Euler Angles
	[׳GBEuc׳, ׳TJEuc׳, ׳QPEuc׳]	Grain-averaged distance from grain boundaries, triple junctions and quadruple points calculated using Dream.3D filters
	[‘Taylor’]	Taylor factor calculated by EVPFFT
	[‘Schmid1’, ‘Schmid2’, ‘Schmid3’, ‘Schmid4’]	HCP Schmid factor for *Basal <a>, Prismatic <a>, Pyramidal <a> and Pyramidal <c+a> respectively*
	[׳min_mis׳, ׳max_mis׳]	Minimum and maximum misorientation between grain and its nearest neighbors
	[׳KernelAvg׳]	Kernel average misorientation in a grain calculated using Dream.3D filters
	[ ׳ph׳]	Phase of grain (=1) for all grains as single phase
	[׳Crss_sch_1׳, ׳Crss_sch_2׳, ׳Crss_sch_3׳, ׳Crss_sch_4׳]	Ratio of CRSS to Schmid factor in for *Basal <a>, Prismatic <a>, Pyramidal <a> and Pyramidal <c+a> slip modes respectively*
	[‘Slip1, ‘Slip2, ‘Slip3, ‘Slip4’, ׳N_Active_slipsys׳]	Slip activity fraction in *Prismatic <a>, Basal <a>, Pyramidal <a> and Pyramidal <c+a> slip modes respectively* calculated using EVPFFT
	[׳AvgC_Axes_0׳,׳AvgC_Axes_1׳, ׳AvgC_Axes_2׳]	Unit vector components describing the HCP c-axis orientation calculated using Dream.3D filters
	[׳r׳, ׳theta׳, ׳phi׳]	Polar coordinates of HCP c-axis w.r.t the sample frame
	[׳NeighborList׳, ׳MisorientationList׳, ׳AvgMisorientations׳]	List of grain neighbors, grain neighbor misorientations and the average misorientation per grain calculated using Dream.3D filters
	[׳EquivalentDiameters׳, ׳FeatureVolumes׳, ׳NumCells׳, ׳Neighborhoods׳, ׳NumNeighbors׳,]	Different measures of grain size calculated using Dream.3D filters
**Directory**		
**PoleFigures**	Contains the processed images representing pole figures for each microstructure	
**Directory**		
**FlowCurves**	Contains the processed images with all the simulated stress strain curves for each microstructure	
**Directory**		
**Code**	Contains the open source jupyter notebook in.HTML format describing the two datasets and how to read them in Python	

Fig. 3 shows the dataset folder structure of the two Mendeley Data archives “Synthetic HCP 3D polycrystalline microstructures with grain-wise microstructural descriptors and stress fields under uniaxial tensile deformation: Part One” and “Synthetic HCP 3D polycrystalline microstructures with grain-wise microstructural descriptors and stress fields under uniaxial tensile deformation: Part Two” located at http://dx.doi.org/10.17632/kt8hfg4t2p.1 [5] and http://dx.doi.org/10.17632/nsfn6tw295.1 [6] respectively

The Pole figures folder consists of images of pole figures of all the microstructures used in this work. The processed data derived from the EVPFFT simulation for the two cases discussed in this article. The grainwise folders contain data derived from the EVPFFT simulation as processed csv files having grain-wise representation of microstructure, microstructural features, equivalent Von Mises stress, strain and slip activity fields. The voxelwise folders contain processed hdf5 files with voxel-wise representation of microstructure, stress, and strain field on a 128 × 128 × 128 grid. The FlowCurves folders contain the stress-strain curve obtained from each EVPFFT simulation. Table 7 further describes the data contained in these files. Note that the numbering convention used for slip activities and Schmid factors is different and care must be taken during analysis.
